# A national strategic plan for reducing the burden of sexually transmitted infections in Israel by the year 2025

**DOI:** 10.1186/s13584-017-0141-8

**Published:** 2017-04-19

**Authors:** Daniel Chemtob, Dan Gandacu, Zohar Mor, Itamar Grotto, Emilia Anis, Elliot Rosenberg

**Affiliations:** 1 Department of Tuberculosis and AIDS, Ministry of Health, Jerusalem, Israel; 2 Division of Epidemiology, Ministry of Health, Jerusalem, Israel; 3 Tel Aviv Regional District Health Office, Ministry of Health & School of Public Health, Tel Aviv University, Tel Aviv, Israel; 4 Public Health Services, Ministry of Health, Jerusalem, Israel; 5 Department of Occupational Health, Ministry of Health, Jerusalem, Israel

## Abstract

**Background:**

There is on ongoing debate in the literature regarding the real burden of STIs (sexually transmitted infections) in Western countries and the proper strategies needed to estimate and to prevent them. Our purpose is to present an evidence-based national strategic plan for STI prevention in Israel through assessing the current burden of illness, leading international preventive strategies, and practical policymaking experience.

**Methods:**

Epidemiologic and health policy data on STIs were analyzed from various sources: a) systematic national surveillance data for the years 2002–2014; b) the international scientific literature (published between 2002–16; keywords: Sexually Transmitted Diseases (STD) (or STI) AND prevention AND intervention AND gonorrhea OR chlamydia OR syphilis; c) internal Ministry of Health (MOH) analyses and reports, and d) expert opinion.

**Results:**

Incidence rates in Israel of *Chlamydia trachomatis* (chlamydia), *Neisseria gonorrhea* (gonorrhea) and *Treponema pallidum* (syphilis) are lower than in most Western countries. However, rates vary among population subgroups: chlamydia, gonorrhea and syphilis are higher in Jews than in non-Jews, and this gap has increased for chlamydia over the past decade. Primary and secondary syphilis rates have increased among men having sex with men (MSM). It is likely that STIs are under-reported and that incidence is even rising due to migration.

A key recommendation is the establishment of an active surveillance system of STIs, utilizing active case finding in high risk populations, along with regular contact with STI clinics run by the four national health management organizations and by the MoH. As with most European countries, the low prevalence of chlamydia and gonorrhea does not justify population-wide screening. Conversely, the increasing incidence of syphilis among MSM should lead to regular screening among this group.

**Conclusions:**

A national STIs prevention strategy for the year 2025 was presented. Although the current burden of illness is low relatively to other Western countries, this is thought to reflect a certain degree of underreporting. These and other gaps suggest a need for focused epidemiologic and health services research to better characterize health risk behaviors as well as provider practice patterns. Innovative implementation strategies have been described, together with the capacity building components needed for developing specific and implementable policy recommendations for the year 2025.

## Introduction

The incidence rates of the three main sexually transmitted infections (STIs) (other than HIV) in Israel- *Chlamydia trachomatis* (chlamydia), *Neisseria gonorrhea* (gonorrhea), and *Treponema pallidum* (syphilis) are lower than those in most western countries [1, 2]. However, these rates may actually be higher due to under-reporting of new cases. Additionally, if appropriate preventive and therapeutic measures are not implemented in a timely fashion, rates may rise due to the following factors: (1) importation of additional cases by migrants from countries with either high prevalence levels of STIs or from countries experiencing recent epidemic outbreaks followed by dissemination within the veteran population; (2) international travel by a native and migrant residents; (3) a significant prevalence of commercial sex work (CSW) in urban areas [2].

Meeting these challenges requires a thorough mapping of the STI epidemiology in Israel, the development of a comprehensive preventive strategy, as well as a practicable implementation program. These challenges have been addressed within the context of a national prevention initiative entitled ‘Healthy Israel 2020’, which aims to increase the life expectancy and the quality of life of Israel’s citizens, while reducing health inequity (specific recommendations for STIs have been postponed to 2025). Topic areas for the entire initiative were selected on the basis of their respective burdens of disease [3]. One of the topic areas covered the prevention of infectious diseases; a subcommittee thereof focused on the prevention of Tuberculosis (TB), HIV, and STIs. Recommendations on the topics of TB-HIV have been published elsewhere [4]. The current integrative article focuses on the development of national targets and objectives for the reduction of the disease burden due to STIs by the year 2025, as well as an evidence-based, and implementable strategic plan to achieve that aim.

## Methods

Data on the epidemiology, preventive interventions and best practice implementation strategies for the prevention of the three main STIs in Israel were analyzed and accessed from various sources. Systematic surveillance data collected by the Division of Epidemiology in the Israeli Ministry of Health (MoH) for the years 2002–2014 was used to describe epidemiological STI data on the Israeli population in addition to internal MOH analyses and reports (“grey literature”). Country-level STI incidence rates were collected from the databases of the World Health Organization (WHO) Regional Office for Europe, the US CDC, and the Australian National Notifiable Disease Surveillance System. Primary and secondary preventive interventions to reduce the burden of STIs published between 2002 and 2016 were accessed from the English language scientific literature by entering the following keywords into PubMed: Sexually Transmitted Diseases (STD) (or STI) AND prevention AND intervention AND gonorrhea OR chlamydia OR syphilis. Twenty one articles describing preventive interventions were retrieved, primarily from European countries and the United States (US) for each specified STI (gonorrhea, chlamydia or syphilis). Particular attention was focused on data/recommendations issued by evidence-based international and other national preventive health oriented organizations such as, the WHO, the Cochrane collaboration, the US Preventive Services Task Force, the Task Force for Community Preventive Services, as well as various European countries.

Throughout our investigation, numerous discussions were held between the MoH staff, several stakeholders (both governmental and non-governmental), and senior medical professionals; these were further elaborated upon by the Healthy Israel 2020 sub-committee on STIs in order to develop a coherent, evidence-based national plan to reduce the burden of STIs in Israel.

## Results

### Epidemiology

#### International

Incidence rates for the three main STIs vary widely between countries and show different trends over time. When analyzing the 53 WHO European countries, many did not report STI incidences annually to the WHO European region [1]. Incidence trends (per 100,000 population) from countries who regularly reported to WHO European region, including Israel, were accessed for the years 2006–2013 [1]. In addition, trends in the US and Australia were also analyzed for this period [5, 6].

##### Chlamydia

Incidence rates (per 100,000 population) in the 27 WHO European region countries which reported data for the year 2012 were highest in the Scandinavian countries; Iceland led this region with 576 cases/100,000. Rates tended to decrease merely from north to south: the United Kingdom - 378, Belgium - 43, Israel - 10.8, and Spain a mere 1.9. The high rates of Chlamydia and discrepancies between Scandinavian countries and other countries are also a partial reflection of ascertainment and reporting biases.

Most of the WHO European region European countries reviewed showed a distinct increase over the years 2006–2013. This increase was noted in other countries as well: the rate in the USA increased from 344/100,000 in 2006 to 443/100,000 in 2013 [5]; and in Australia, increased from 230/100,000 in 2006 to 363/100,000 in 2013 [6].

##### Gonorrhea

Incidence rates (per 100,000 population) in the 34 WHO European region countries which reported data for the year 2012 were much lower than those of chlamydia, ranging from 45.5 in the United Kingdom and 36.5 in the Russian Federation, to 0.45 in Italy, 0.16 in Montenegro and 0.11 in Bosnia [1]. Many countries experienced fluctuations during the years 2006–2013. Israel’s rates fluctuated between 2.1 and 4.1/100,000 during this period. US rates decreased from 2006 to 2009, but then began to rise again. US rates were significantly higher than those in the European region, reaching 105.3/100,000 in 2013 [5]. Australia has also experienced fluctuating rates, with 41.6/100,000 in 2006, 35.7/100,000 in 2008, but rising to 64.5/100,000 in 2013 [6].

##### Primary and secondary early syphilis

Incidence rates (per 100,000 population) in the 25 WHO European region countries which reported data for the year 2012 were all of the same order of magnitude, ranging from 11.9 in Georgia to 1.3 in Sweden [1] (except the Republic of Moldova which reported an exceptionally high rate of 64.7). Israel, though, reported an even lower rate: 0.9/100,000. Since 2006, slightly more countries have noted an increase rather than a decrease in incidence trends, but in most they fluctuated. The US rate of 5.5/100,000 in 2013 is higher than that seen in almost all European countries [5]. Australia has experienced fluctuating rates, with 4.3 in 2006 rising to 6.8 in 2007, dropping to 5.1 in 2010, but then rising again to 7.6 in 2013 [6].

On the basis of current passive surveillance, incidence rates of chlamydia, gonorrhea and syphilis are low in Israel relative to northern European countries [1], the USA [5] and Australia [6], and are roughly similar to those reported by southern Mediterranean European countries.

#### Israel: an in-depth analysis

**Fig. 1:**
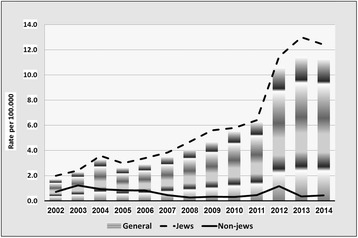
Incidence of *Chlamydia trachomatis* infection, by year and by population groups, Israel 2002–2014

**Fig. 2:**
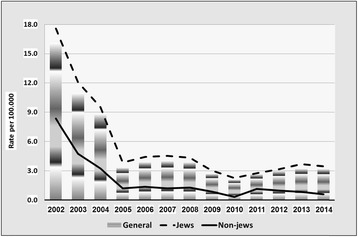
Incidence of *Neisseria gonorrhea* by year and by population groups, Israel 2002–2014

**Fig. 3:**
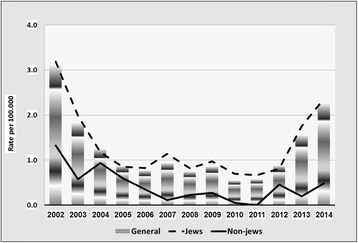
Incidence of *Treponema palladium* Infections by year and by population groups, Israel 2002–2014

In 1994, Chlamydia was added to the list of STIs already defined in Israel as reportable diseases. STI incidence rates by population groups (for the period 2002–2014) appear in Figs. 1, 2 and 3.

The main findings were as follows:

- Rates of chlamydia are appreciably higher in Jews than in non-Jews. This difference has increased over the past decade. Overall, rates have markedly increased in recent years (from 2.9/100,000 in 2006 to 11.3/100,000 in 2014).
- Rates of gonorrhea have decreased in the last decade, and are approximately three times higher in Jews than in non-Jews. Males account for the majority of the cases (male to female ratio >4). Increasing requests for bacteriologic testing of pharyngeal specimens, especially in men having sex with men (MSM) and in CSW, may indicate an increasing trend in the transmission of pharyngeal gonorrhea [7].
- The rates of syphilis are roughly three to four times higher in Jews than in non-Jews. Between the years 2002–2011, rates have dropped by over 70% in the Jewish population and by 80% in the non-Jewish population, before increasing back since 2012 and forming a U-curve, especially prominent among the Jewish population. This recent increase is largely influenced by an increase of reported new male cases, which are assumed to be mostly among MSM, the group at the greatest risk of infection in recent years [8].
- The number of cases of syphilis among HIV-infected MSM increased from 0 to 2 cases/year in the 1990–2000 period to 10–18 cases/year during the 2005–2009 period. In the Tel Aviv district, where 90% of the patients were MSM, the number of males infected with syphilis increased from 5 cases in 2005 to 40 cases in 2009. In the MoH STI clinic in Tel-Aviv, 1,064 (22%) MSM and 3,755 (78%) heterosexuals were tested. Positivity rates for HIV, urethral *N. gonorrhea* and infectious syphilis in MSM were higher than in heterosexuals (2.5%, 2.5%. 0.7% vs. 1.6%, 1.3%, 0.3%, respectively), while urethral *C. trachomatis* was higher in heterosexuals than in MSM (2.7 vs. 1.4%, respectively) [9].
- At National level, the peak age of incidence of all three STIs was 15–44, where the 25–34 year old age group suffered the greatest burden of infection. Further analysis of incidence trends in these three major STIs by age group (15–24; 25–34; 35–44) appears in a recent publication [2].

### Setting the 2025 objectives and targets

The aforementioned data sources resulted in the generation of the following baseline incidence rates for the three STIs of interest in 2014:

- Chlamydia: 11.3/100,000
- Gonorrhea: 3.2/100,000
- Primary and secondary syphilis: 2.3/100,000

The current Israeli STI surveillance system is predominantly passive and therefore the generated data is inherently incomplete due to partial reporting and capture of disease incidence data. Conversely, when setting target goals and national objectives to decrease the rates and/or the burden of disease, it is critical to establish more authoritative baseline values. These limitations led to the decision by the authors of this article to defer the definition of the 2025 objectives and target values until more accurate baseline values could be generated.

### Interventions

#### Primary prevention

A. Evidence of effectiveness
  1. Clinically-based
    High intensity behavioral counseling interventions (contact time of over 2 hours) targeted to sexually active adolescents and adults at increased risk for STIs reduced the incidence of STI’s when assessed 12 months post-counseling [10].
    Note: Adults at increased risk included those with current STIs or infections within the past year, or those with multiple concurrent sexual partners, and adults who did not consistently use condoms.
  2. Community-based
    2.1 Comprehensive risk reduction (CRR) interventions delivered in schools or in community settings to groups of adolescents (aged 10–19) were effective when including one of the following approaches, as appropriate (and may include components such as condom distribution and STI testing):
      Propose several behaviors, including sexual risk reduction strategies, but suggest abstinence as the behavior of choice;
      Recommend both abstinence and sexual risk reduction as equivalent strategies;
      Advocate sexual risk reduction strategies as a sole or at least as a primary strategy [11].
    2.2 Community-based youth development behavioral interventions coordinated with community service to reduce sexual risk behaviors in adolescents
      These interventions address a broad range of health and wellness issues. They may not necessarily include components that are focused directly on pregnancy and STI prevention. Key components include social, emotional, or cognitive competence training that promotes pro-social norms, improved decision making, self-determination, and communication skills. These interventions serve to strengthen positive bonding experiences between youth and their peers or non-parental role models.
      The community service component is broad. Scheduling a variety of activities in community settings such as nursing homes, hospitals, and homeless shelters would be appropriate [12].
      Community and school-based interventions are resource intensive. Before implementing such a program, cost-effectiveness analysis and small-scale piloting within communities would be necessary.
B. International recommendations
  WHO
    The World Health Assembly endorsed the global strategy for the prevention and control of STIs in May 2006. The strategy urges all countries to control the transmission of STIs by implementing a number of interventions, including the following [13]:
    Involvement of all relevant stakeholders, including the private sector and the community, in prevention and care of STIs;
    Specific services for populations with frequent or unplanned high-risk sexual behaviors - such as Commercial Sex Workers (CSW), adolescents, military personnel, substance users and prisoners;
    Prevention by promoting safer sexual behaviors;
    General access to quality condoms at affordable prices or free of charge for specific populations.
  US
    The US Preventive Services Task Force (USPSTF) recommends high-intensity behavioral counseling to prevent STIs for all sexually active adolescents and for adults at increased risk for STIs.
      Grade: B Recommendation [10].
    The Community Preventive Services Task Force (CPSTF) recommends both CRR interventions delivered in school or in community settings to groups of adolescents [11], as well as youth development behavioral interventions coordinated with community service [14].
  Israel
    The Israeli Task Force on Health Promotion and Disease Prevention (a branch of the Scientific Council Israeli Medical Association) recommends for all 13–19 year olds to be counseled in matters regarding sexual behavior, STIs and contraceptive use. This is optional for 20–39 year-olds. No information is provided about the manner (methodology, training, frequency, duration etc.) in which this counseling is to be performed [15].

#### Secondary prevention (screening)

A. Evidence of efficacy
  1. Chlamydia
    Two randomized controlled trials demonstrated a reduction in medical complications in women members of a Health Maintenance Organization (HMO) and in a high school that implemented Chlamydia screening [16, 17]. A recent summary article from the US CDC justified screening sexually active young, as well as high risk older females, due to the large and costly burden of preventable illness (presenting as pelvic inflammatory disease (PID) and its sequela, tubal infertility), the asymptomatic nature of the infection in females, the ease of diagnosis with nucleic acid amplification tests, the highly efficacious treatment options, and the randomized trial data showing a reduction in PID incidence following screening [18].
    Conversely, a systematic review did not find evidence for even opportunistic screening of women younger than age 25, if not considered as high risk [19]. A Cochrane review found Chlamydia screening to have only a modest effect in reducing PID risks at the individual level, but had no effect on epididymitis or on infection levels among in men and women [20].
  2. Gonorrhea
    Indirect evidence shows that screening sexually active women age 24 and younger, as well as older women at increased risk of infection (those with a new or more than one sexual partner, a sexual partner infected with an STI, inconsistent condom use, a history of previous or coexisting STIs, or those exchanging sex for money of drugs) may prevent other complications associated with gonococcal infection, such as pelvic inflammatory disease and its sequelae [21].
  3. Syphilis
    Screening tests for syphilis can adequately diagnose the disease. Effective and cheap antibiotic treatment is also available for cure. High risk individuals (including MSM, CSW, and adults in correctional facilities) have a higher pre-test probability of being diagnosed with syphilis. Despite the above, there are insufficient data to conclusively prove that screening reduces syphilis-related morbidity [22].
    The recommendation to screen pregnant women is based upon observational evidence that screening decreases the proportion of newborns with clinical manifestations of syphilis infection and those with positive serology [23].
B. Cost-Effectiveness
  In a review of 55 cost-effectiveness studies focused primarily on Chlamydia and HIV interventions, one-on-one interventions such as counseling (and screening) were proven to be cost-effective [24]. An editorial on the article [25] noted that these cost-effectiveness calculations were conservative because the benefits of STI prevention such as reduction of HIV incidence and productivity losses were excluded. A cost-utility analysis of Chlamydia screening calculated that the incremental cost-effectiveness ratio relative to the next most effective strategy would cost less than $25,000 USD/QALY for annual screening followed by semi-annual screening for those with a history of infection, thus classifying it as very cost effective [26]. This has been corroborated by a more recent United Kingdom (UK) calculation based on modeling: it was estimated that it would cost £506 (=US $776 - Conversion rate as of April 15, 2013) per infection treated [27]. Israeli cost-effectiveness data is not yet available.
C. Screening policies in selected western countries
  US
    Chlamydia and Gonorrhea
      The USPSTF recommends that all sexually active women aged 24 and younger, as well as for older women at high risk for STI be screening for chlamydia and gonorrhea. High risk is defined as those with one of the following risk factors: those with a history of previous or concurrent STI, those with a new or more than one sexual partner, a sex partner who is currently infected with STI, sporadic (inconsistent) use of condoms, and those who exchange sex for drugs or money.
        Grade: B recommendation [21].
    Syphilis
      The USPSTF strongly recommends that persons at increased risk for syphilis infection be screened by clinicians. These include MSM engaged in high-risk sexual behavior, CSW, persons who exchange sex for drugs, and those in adult correctional facilities.
      Grade: A Recommendation [28].
      The USPSTF strongly recommends all pregnant women be screened for syphilis infection.
      Grade: A Recommendation [29].
  EU (European Union)
    Chlamydia
      In guidance presented in June 2009, The European Centre for Disease Prevention and Control (ECDC) recommended a step-by-step chlamydia control strategy whereby primary prevention, case management, and opportunistic testing to specified sub-populations attending clinical services be carried out, and evaluated, alongside the development of both patient management infrastructures, and quality controls before population-based screening be enacted [30].
      Most European countries lack a national screening program for Chlamydia for asymptomatic individuals [30]. A national Chlamydia screening program was put into place in the UK in 2007 [31] for women under age 25 attending various clinical and non-clinical settings (e.g., universities and sporting events). A pilot program of annual postal invitation was introduced in three regions of the Netherlands in 16–29 year-olds in early 2008 [30]. A register-based screening program using mailed home-collected specimens is planned in Norway [30]. Opportunistic screening is widespread in Sweden but it lacks national coordination and is conducted on a county basis. Several northern European countries perform opportunistic testing of asymptomatic individuals, e.g., Denmark tests people with frequent sex partner change and women aged 25 and under before an IUD insertion (although in two of sixteen communities in the country proactive screening has been introduced - via postal invitation) [30]. Iceland screens all women presenting for termination of pregnancy and egg or sperm donors [30]. Canada recommends population-wide screening [32]. Since 2005, Australia has been moving in this direction [33].
    Gonorrhea
      UK (United Kingdom) [34]
        There is no basis to support widespread, unselected screening for gonorrhea where only meager evidence for selective community screening exists in the UK. STI data is limited for those treated outside the genitourinary medical (GUM) clinics and prevalence studies are rare. The prevalence of gonorrhea infection varies widely between and within communities and patient populations. Gonorrhea diagnoses and subsequent complications are infrequent compared to chlamydia. Higher prevalence of infection than the general population is found among inner-city residents, STIs clinics attendees, military personnel, prisoners and MSM. The immediate health benefits from an accurate gonorrhea diagnosis are the subsequent reduction of HIV transmission or acquisition risk, support such an intervention. However, the health benefits must be weighed in light of the cost and adverse effects of screening. Localized interventions targeted at the core high-risk groups are likely to be more cost-effective and beneficial than unselected community-wide screening.
      Syphilis [35]
        Routine tests for syphilis should be taken in all pregnant women, those donating blood, and in the following high risk groups for syphilis infection: (a) all patients who are newly diagnosed with a STI; (b) persons infected with HIV;(c) patients with hepatitis B or C; (d) patients suspected of early neurosyphilis (i.e., unexplained sudden visual loss [uveitis], unexplained sudden deafness [otitis] or meningitis); (e) patients who engage in sexual behavior that puts them at risk (e.g. MSM, CSW, and all those individuals at higher risk of acquiring STIs).
  Israel
    1. Israeli Task Force for Health Prevention and Disease Prevention [15]:
      All pregnant women should be tested for syphilis (with the VDRL - Venereal Disease Research Laboratory - test or by ELISA - enzyme-linked immunosorbent assay). Serologic testing should be considered for pregnant women that are high-risk for Chlamydia and no recommendation was issued regarding screening women for gonorrhea.
    2. Israeli Ministry of Health
    Chlamydia and gonorrhea
      Due to the relatively low incidence of chlamydia and gonorrhea in Israel, a population-wide screening program is unjustified. Continued prevalence surveys for specific high-risk populations and various population substrata will be used to guide future policy decisions. Evidence-based routine surveillance and case management should continue.
    Syphilis
      Screening is recommended for the following high risk populations:
      - Pregnant women
      - MSM
      - Persons who exchange sex for drugs and other CSW

Rationale: these recommendations dovetail with USPSTF recommendations [28, 29] except with regard to adults in correctional facilities. According to Israeli correctional facility health reports (Aurkin-Tischler D, Israeli Prison Services. Personal communication, 2012), screening is not required because syphilis does not pose a serious threat to this population.

## Discussion

Conservative MoH policy regarding STI screening in Israel reflects the relatively low national incidence of STIs, although underestimation is almost certainly an important issue, similar to many countries [36, 37]. Underestimation results from passive surveillance systems’ inability to capture true population STI incidence, most likely originating from structural and functional limitations, and threatens accurate reporting due to the intrinsic biases of these systems. Therefore we recommend implementing measures to optimize the existing passive surveillance system including supplementing it with a robust, active surveillance system (as has already been accomplished for HIV in Israel [38]).

### Developmental data objectives and capacity building

- Active surveillance
  Active surveillance projects should be undertaken in high risk population groups such CSW, IVDU (intravenous drug users), MSM, and HIV carriers, to rigorously establish the burden of disease in each subpopulation and be performed in sentinel locations such as STIs clinics, dedicated MSM and CSW venues.
- Passive surveillance
  The Israeli passive surveillance system should be revamped by utilizing the following methods:
  A. Increased capacity building
    Additional professional personnel should be recruited by the MoH TB and AIDS department to enable it to also handle non-HIV STIs. This will facilitate improvements in STI surveillance and data processing, as well as other important tasks (e.g., development of field interventions and evaluation of such programs). Combined HIV and other STI surveillance, along with other activities has been done by several EU countries, such as France and the Netherlands (Caroline Semaille, from the “Institut de Veille Sanitaire” of France - and Eline Op de Coul, from the Centre for Infectious Disease Control of The Netherland, April 2012, Personal communications).
  B. Explore the adequacy of STI clinic health service coverage and staff data collection roles
    To determine the need for possible modification and improvements to existing services, population coverage of existing STI clinics, access thereof, and role definition of the staff may be further examined.
  C. Improve the accuracy of clinical diagnoses
    Regularly update professional guidelines to reflect the state of the science;
    Establish a uniform, computerized case investigation form for use by epidemiologic nurses;
    Provide a specific training course for STI clinic healthcare personnel: physicians, nurses, social workers, and train them to effectively interview patients and to improve epidemiological investigations;
    Increase the awareness of community-based primary care physicians and hospital staff of the need to culture patients with venereal secretions prior to initiating empiric treatment regimens;
    Ensure that PCR diagnostic tests are available in all community clinics to diagnose chlamydia and gonorrhea;
    Provide health providers with suitable equipment for sample collection, and increase their awareness of the proper methodology for sample transport and delivery;
    Improve laboratory assessment of antibiotic resistance in positive cultures, and assure timely notification of providers about specific diagnoses (as well as about the latest trends in microbial resistance in order to facilitate effective treatment);
    Authorize requests for Nucleic Acid Amplifications Test (NAAT) testing by primary care physicians nationwide, rather than requiring case-by-case approval by urologists;
    Enhance the diagnostic capability of the National STI’s Laboratories (per disease);
    Consider data analysis using tools such as GIS [2].

### Developmental interventional objectives

- Behavioral surveillance and research should be expanded to better describe population risk behaviors and attitudes towards safe-sex to develop more effective interventions. Research indicates a relatively low awareness of STIs among primary health care clinicians [39]. Barriers and facilitators of clinician adherence to guidelines should be explored. The effectiveness of counseling should be assessed.
- Improve contact tracing: Target populations of TB and STI programs are often similar and both utilize similar public health strategies. Rothenberg and others [40] have suggested expanding the contact tracing approach used by the national TB programs to STI programs (i.e., implementing horizontal integration of these programs). Innovative strategies such as identification of social networks should be adopted [2] that can be especially timely during periods of financial constraints [40]. Israel does not (yet) have a national STI program, but a national TB program has existed since 1997 [41]. However, implementing this scheme will require extensive planning (including development of appropriate training) to adapt these techniques to the specific needs of community health work to develop a focused contact tracing program [2].
- A sexual health initiative may be progressively developed and implemented jointly by the Ministries of Health, Education, and Welfare. A recent example is Australia’s Second National STI Strategy 2010–2013 [42].
- Involve and empower additional health providers who treat potential patients. These include urologists, gynecologists, dermatologists, family physicians, and emergency room medical staff. Special effort should be invested to involve and continually update independent practitioners unaffiliated with the major HMOs.
- The current AIDS national steering committee should be expanded to include several new members specializing in STI.
- We also recommend performing cost-effectiveness analyses related to different components of future interventions as the second phase of this program.

## Conclusions

A national STI (chlamydia, gonorrhea, and syphilis) prevention strategy for the year 2025 is presented. Although the current burden of STIs is relatively low compared to other Western countries, this is thought to be a partial result of underreporting. These and other gaps suggest a need for focused epidemiologic and health services research utilizing strategies such as active surveillance and field surveys to better characterize health risk behaviors as well as provider practice patterns. Primary preventive counseling is recommended for adolescents and younger adults. Due to the low prevalence of chlamydia and gonorrhea, only case finding in high risk populations is recommended. Screening with the VDRL test or the ELISA test is suggested for pregnant women, MSM, those exchanging sex for drugs, and CSW. Innovative implementation strategies include: cross-training of existing MoH TB personnel, enhanced training and interface with community clinical specialists, and procurement of more advanced diagnostic and analytic tools at all levels of care, in conjunction with expanded authorization of healthcare personnel to avail themselves of these tools.

Implementation of these recommendations will require a commitment to reasonably increase the capacity of the Department of TB and AIDS and satellite organizations including increased staffing, development of specific training modules for infectious disease and primary care professionals, and major improvements of the existing technical infrastructure, including improvement in efficient and overall quality of laboratory diagnostic capabilities.

This should provide a strong foundation upon which to develop specific and implementable national STI prevention policy recommendations by 2025.

## Abbreviations

Chlamydia: *Chlamydia trachomatis*
CPSTF: Community Preventive Services Task Force
CRR: Comprehensive risk reduction
CSW: Commercial sex work
ECDC: European Centre for Disease Prevention and Control
ELISA: Enzyme-linked immunosorbent assay
EU: European Union
Gonorrhea: *Neisseria gonorrhea*
GUM clinics: Genitourinary medicine clinics
HIV: Human Immunodeficiency Virus
HMO: Health Maintenance Organization
IVDU: Intravenous drug users
MoH: Ministry of Health
MSM: Men having sex with men
NAAT: Nucleic Acid Amplifications Test
PCR: Polymerase chain reaction
PID: Pelvic inflammatory disease
STDs: Sexually transmitted diseases
STIs: Sexually transmitted infections
Syphilis: *Treponema pallidum*
TB: Tuberculosis
UK: United Kingdom
US: United States of America
USPSTF: US Preventive Services Task Force
VDRL test: Venereal Disease Research Laboratory test
WHO: World Health Organization
